# Acoustic Correlates of English Lexical Stress Produced by Chinese Dialect Speakers Compared to Native English Speakers

**DOI:** 10.3389/fpsyg.2022.796252

**Published:** 2022-03-08

**Authors:** Xingrong Guo

**Affiliations:** College of Foreign Languages, Shanghai Maritime University, Shanghai, China

**Keywords:** English lexical stress, acoustic correlates, transfer, Chinese dialects, production

## Abstract

English second language learners often experience difficulties in producing native-like English lexical stress. It is unknown which acoustic correlates, such as fundamental frequency (F0), duration, and intensity, are the most problematic for Chinese dialect speakers. The present study investigated the prosodic transfer effects of first language (L1) regional dialects on the production of English stress contrasts. Native English speakers (*N* = 20) and Chinese learners (*N* = 60) with different dialect backgrounds (Beijing, Changsha, and Guangzhou dialects) produced the same stimulus including both trochaic and iambic patterns. Results showed that (a) all participants produced the stressed syllable with greater values of F0, duration, and intensity; (b) Native speakers of English employed an exquisite combination of F0, duration, and intensity, while the dialect groups transfer their native prosody into their production of English lexical stress, resulting in the deviation or abnormality of acoustic cues. Results suggest that L1 native dialect background is considered as a potentially influential factor which may transfer in L2 speech encoding and decoding process.

## Introduction

English is very important in speech communication. First language (L1) typological factors have been reported as determining, to a degree, the production and perception success rate for second language (L2) stress (Altmann, 2006). Trubetzkoy (1958) claimed that L2 perception is “filtered” by the L1 “sieve.” The effects of such filtering on production are most apparent when a L2 speaker is perceived as showing a foreign, or non-native, accent, which is largely detectable due to L1 transfer. Prior research has revealed that L2 learners of non-stress languages, such as Chinese, may not process stress as a native does (Wang, 2008; Zhang et al., 2008; Qin et al., 2017). L1 lexical tone sensitivity, for L1 language speakers, such as Cantonese, for L2 speakers, or even children, might contribute either directly or indirectly to L2 English lexical stress sensitivity (Choi et al., 2017).

Contrastive Analysis Hypothesis (CAH; Lado, 1957) proposes that similarities and differences between L1 and L2 may result in positive and negative transfers. The “tendency to transfer is especially powerful in second language acquisition” (Major, 2001). Transfer can occur in several linguistic dimensions, including the lexical, syntactic, and phonological domains (Major, 2001). Phonologically, L1 interference may occur at segmental and suprasegmental levels (Shen, 1990). L1 prosody significantly influences L2 acquisition. The Stress Typology Model (STM; Vogel, 2000; Altmann, 2006) predicted that for production, a setting of “stress language” (e.g., English) parameter in the typological hierarchy branching would lead to a more native-like L2 stress placement, otherwise will cause disadvantages for non-stress languages (e.g., Chinese) speakers (Altmann, 2006).

Cross-linguistic research on English lexical stress acquisition has been an issue of great interest since the 1950s (Fry, 1955; Lieberman, 1959; Altmann, 2006). Despite myriad studies have been carried out in this field over the past decades. Most findings are generally regarded on behalf of speakers’ L1 as a whole. L1 dialect effect has been neglected. Recent research has begun to address this neglect, suggesting that second language acquisition research should attend to L1 dialect influence. O’brien and Smith (2010) noted that one shortcoming of the prior research on L2 acquisition has been that researcher in phonetics has highly presumed the levels of homogeneity of participants and ignored the influence of different dialects.

Grabe (2002) has argued that phonological research may be hampered if actual speech variability was not fully investigated. Altmann (2006) has claimed that it was necessary to design special experiments to explore more subtle differences existing in learners’ L1 dialects in stress production and perception. According to Bai (2001), Chinese speaker utterances of English may be affected by various L1 dialect factors, such as syntax, semantics, phonology, stylistics, and stratification. In addition to the impact of Putonghua (Mandarin), L2 speakers’ L1 accent tends to have an influence on the production of L2 tone and intonation features.

Previous work on English stress has proved that Mandarin and English speakers show different preferences in using prosodic cues in both stress production (Chen et al., 2001; Zhang et al., 2008) and perception (Wang, 2008; Zhang and Francis, 2010). Most of the research has focused on comparing the performance of Chinese learners and Native English speakers. Those results generally treated Mandarin speakers as represent Chinese speakers as a whole. Nevertheless, learners’ dialect background was overlooked or obscured.

Relatively few studies have attended to the interplay of the acoustic correlates of English lexical stress by Chinese learners of English, especially from the perspective of diverse dialects, which have largely been neglected. How L1 dialect specific prosody gets transferred to English stress acquisition, lexical stress in particular, merits more extensive study. The present study is intended to address this gap by examining the possible L1 dialect effects on the production of English lexical stress by speakers with different L1 dialect backgrounds.

### Acoustic Correlates of English Stress

English lexical stress primarily involves an emphasis on individual syllables in a polysyllabic word (Archibald, 1993). It is usually manifested by fundamental frequency (F0), duration, and intensity (Fry, 1955, 1958; Lehiste, 1970). Stressed syllables are usually produced with relatively higher F0, greater intensity, and longer duration compared to unstressed syllables (Goffman and Malin, 1999).

There has been a lack of consensus on the hierarchy of the acoustic correlates of lexical stress in English. Fry (1955, 1958) conducted a series of classic research to explore the influence of the acoustic correlates in English stress perception tasks by native speakers. Fry (1955) found that “when the stress was shifted from the first to the second syllable the most marked variations took place in the relative duration and intensity of the ‘vowel’ portions of the speech wave, while other parts of the wave remained remarkably constant in these respects.” Vowel duration and intensity were found to be most correlated with perceived stress. Fry (1958) investigated the influence on stress placement when duration, intensity, and F0 were manipulated. Results showed changes of vowel duration ratio influenced the listeners’ stress judgments. Intensity ratio also shows a similar effect but it did not cause a complete shift of stress judgment. F0 shows an all-or-none effect, and “the magnitude of frequency change seems to be relatively unimportant while the fact that a frequency change has taken place is all-important” (Fry, 1958, p. 151). Lieberman (1959) demonstrated different findings. He agreed with Fry (1958)‘s finding that F0 is the most relevant cue, but he found that intensity plays a more important role than duration.

Bolinger (1965) considered that in English stress, duration is an “auxiliary and residual cue” and intensity is “negligible both as a determinative and as a qualitative factor.” Lehiste (1970) also concluded that F0 plays a very important role in English stress and intensity is a weak cue. Compared to stressed syllables, English monophthongs were reported to be 50% shorter in unstressed syllables (Crystal and House, 1988). Sluijter and Van Heuven (1996) found that duration plays the most important role, while “F0 and overall intensity have little or no cue-value.” Tong et al. (2015) considered that spectral balance is the most robust cue of English stress. Although researchers disagree on the weight of acoustic correlates of English stress, they all agree that stress is “not a single mechanism” and consists of three main cues (F0, duration, and intensity; Wang, 2008).

Evidence to date suggests that L2 learners are adept at using acoustic correlates in L2 if the same correlates are actively used in L1. Ueyama (2000) reported that beginning-level L2 speakers tended to import active L1 prosodic characteristics (e.g., F0 in Japanese) to L2 learning, and they learned to control the inactive acoustic cue (e.g., duration in Japanese) in the L1 system. Nguyen and Ingram (2005) found that the active role of L1 tonal cues (F0 and intensity in Vietnamese) facilitated producing English lexical stress (F0, intensity). In their study, beginners failed to encode the inactive cue (duration), but the advanced speakers could produce native-like duration contrasts. However, Keyworth (2015) argued that native-like command is attainable through increased study or L2 input. Failure to properly produce these cues may contribute to a foreign accent.

### Acoustic Correlates of Chinese Dialects

Languages can be classified into stressed-timed or syllable-timed (Pike, 1945). English is a typical stressed-timed language. Chinese is usually labeled as syllable-timed. Miller (1984) proposed a compromise classification that there exists a continuum between syllable- and stress-timing. Xu (2008) agreed with Miller’s proposal and rated Chinese rhythms as ranging from stress-timed (e.g., Beijing dialect) to syllable-timed (e.g., Cantonese) from north to south.

Altmann (2006, p. 45) described that “duration is the primary cue for stress” in English. In a non-stress language, such as Mandarin Chinese, “we do not observe the combination of pitch, duration and intensity referred to previously as the manifestation of (word) stress. Instead, pitch alone typically provides crucial word level contrasts.” Altmann (2006, p. 46).

Chinese is classified into seven major dialect groups: Mandarin, Wu, Gan, Xiang, Min, Kejia (Hakka), and Yue (Cantonese; Yuan, 1989). This study selected Beijing (Beijing Mandarin), Changsha (Changsha Xiang), and Guangzhou (Guangzhou Cantonese) dialect speakers to test whether dialectal differences existed in producing English lexical stress compared to American English (AE) speakers.

The three main acoustic correlates of Chinese tones were F0, duration, and intensity. Acoustic cues of tones in Chinese are not as controversial as in English stress. However, the weight of acoustic cues is different. F0 is the most important acoustic parameter of Chinese tone (Howie and Howie, 1976; Lin, 1988; Fu and Zeng, 2000). Lin (1988) conducted an experiment with synthesized speech, and he proved that F0 was the primary cue for tone. He found that any variation in duration or intensity would not affect the tone perception under the circumstance of changing F0 contour into a constant F0. Besides, both F0 height and F0 contour were proved to play important roles in tone perception. Mandarin speakers were found to rely more on F0 contour (Gandour, 1984).

The neutral tone in Beijing and Changsha dialects occurs at the final position of a word and is produced in a light and short way. The syllable-timing of Cantonese was much stronger than Mandarin, since Cantonese has a simple syllable structure without lexical stress and phonological vowel reduction (Mok, 2009). All Cantonese syllables are stressed (Perry et al., 2009). Duanmu (2007) found that full syllables (syllables with lexical tones) in Chinese are equal to stressed syllables in English, while light syllables (neutral tone) are equal to unstressed syllables in English.

### Beijing Dialect

Beijing dialect is phonologically similar to Standard Mandarin. It has four basic tones (T55, T35, T214 and T51; Chao, 1948). The neutral tone (i.e., the unstressed syllable) occurs frequently in the Beijing dialect (Mok, 2009). Lee and Zee (2008) found that (1) the “F0 contour of the neutral tone on the non-initial syllable” was determined mainly “by the preceding citation tone”; (2) of the neutral tone’ intensity “co-varies with the corresponding F0 contour”; (3) the duration of the neutral tone was usually shorter than “the preceding or following citation tone”. Liu and Samuel (2004) mentioned that F0 was the primary acoustic cue for tone perception.

### Changsha Dialect

Changsha dialect belongs to New Xiang dialect. Analogous to Beijing dialect, duration, F0, and intensity are essential acoustic cues of the stressed syllable in Changsha dialect. However, there are a few differences between Beijing dialect and Changsha dialect in weighting the acoustic cues. Changsha dialect has two more lexical tones than Beijing dialect and usually shows lower F0. Unlike the Mandarin tone, F0 does not play the most influential role in Changsha dialect; instead, duration is more crucial than either F0 or intensity (Yi, 2007). Yi (2007) investigated the metrical stress in Changsha dialect and found that its main acoustic cue for stressed syllables was duration, and the duration value of stressed syllables was almost 20–60% longer than that of unstressed syllables. Furthermore, F0 weighed less than duration in discriminating stressed and unstressed syllables. The F0 contour of unstressed syllables usually displayed a downward trend. Intensity and vowel quality did not discriminate stress contrasts in Changsha dialect. For instance, the unstressed syllables might display higher intensity. Neutral tone occurs at the final position of a word and is light and short. This results in the neutral tone being regarded as analogous to an unstressed syllable.

### Guangzhou Dialect

Guangzhou dialect is deemed to be standard Cantonese. Ciocca et al. (2002) found that F0 plays as the “primary, and perhaps sole” role in Cantonese tones. According to Li (1990), only 21.5% characters have the same pronunciations between Cantonese and Mandarin in *Basic Vocabulary Table of Modern Chinese Characters*. Only 23.1% of all the Cantonese words have equivalents in Mandarin (Rao et al., 1997). The rate is very low at 1.78%, when the two colloquial expressions are compared (Zeng, 1982). Therefore, Cantonese and Mandarin are widely considered to be two distinct languages in bilingual studies (Cai et al., 2011; Liu et al., 2017). Guangzhou dialect has nine tones, which does not have the neutral tone. Guangzhou dialect is considered more syllable-timed than Beijing dialect (Mok, 2009). This difference reasonably suggests the hypothesis that Beijing speakers might perform better in duration in unstressed syllables than Guangzhou speakers. Every Cantonese syllable carries a tone without a neutral tone (Bauer and Benedict, 1997). Cantonese and Mandarin, which are both strong syllable-timed and weak syllable-timed, respectively, would result in stress production with differences in terms of duration and intensity (Lin et al., 2013).

### F0 Contours for Tones in Beijing, Changsha, and Guangzhou Dialects

**Figure 1:**
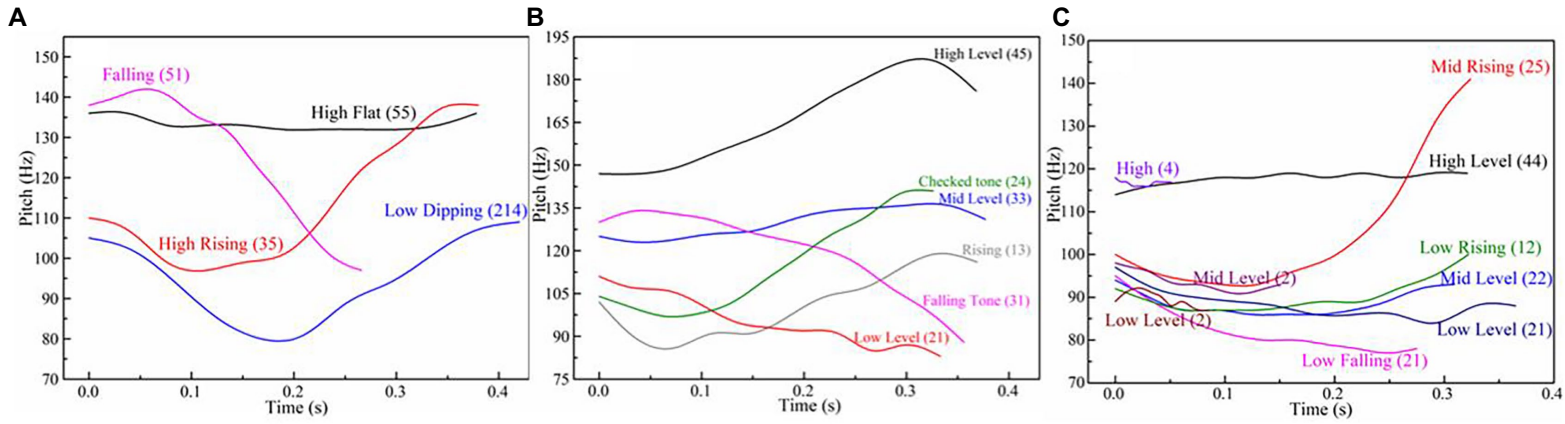
F0 contours for tones in Beijing dialect **(A)** produced by a male speaker on the syllable /ma/, Changsha dialect **(B)** produced by a male speaker on the syllable /pa/, and Guangzhou dialect **(C)** produced by a male speaker on the syllable /si/.

Figure 1 depicts the F0 contours of the four Beijing dialect tones, six Changsha dialect tones, and nine Guangzhou dialect tones. The numbers in parenthesis indicate the relative starting and ending pitch of each tone on a 1–5 scale, with 1 referring to the lowest pitch of the speaker and 5 to the highest pitch (Chao, 1948). The *T* value was calculated according to the formula: $T=\frac{\log x-\log b}{\log a-\log b}\times 5$ (lg x: the observed F0 value in Hz; lg a: the max F0 value of the speaker in Hz; and lg b: the min F0 value in Hz).

Though there are considerable variations in lexical tone production, the basic F0 patterns of the lexical tones are rather consistent at a more abstract level. Since these tones are produced by learners with different dialect backgrounds, it is impossible to compare the absolute F0 levels between different dialects. However, the relative F0 levels can be compared.

### Chinese Speakers’ Production of English Stress

Native Mandarin speakers and Native English speakers rely on different acoustic correlates for English stress production (Lai, 2008). Mandarin relies on lexical tones to distinguish lexical meaning. F0 is “the primary acoustic cue for Mandarin tones,” but duration and intensity “vary consistently across lexical tone categories” (Zhang et al., 2008). Duration and intensity also play important roles, especially in contributing to the perception and categorization of lexical tones (Whalen and Xu, 1992). They can be statistically modeled for tonal representation (Chen, 2015).

Mandarin speakers also used F0, duration, and intensity when producing English stress. However, these cues are used in different ways. Chen et al. (2001) found native Mandarin speakers produced significantly higher F0 and shorter duration in stressed syllables, and higher F0 and greater intensity in unstressed syllables than native Mandarin speakers when producing English sentence stress. Zhang et al. (2008) reported native Mandarin speakers employed native-like intensity and duration for English-stressed syllables but produced higher F0 than native Mandarin speakers. Ng and Chen (2011) found Cantonese speakers proficiently used intensity and duration to signal sentence stress but exhibited consistently higher F0 than native Mandarin speakers. In short, the problems that Chinese speakers face in learning English stress were mainly assumed to arise from tonal transfer. Failure to accurately produce these cues may contribute to a non-native accent.

Some studies suggest that the phonetic realizations of stress may vary across dialects. For example, Bethin (2006) reported that changes across East Slavic dialects revealed a typology of stress and tone. Qin and Tremblay (2014) and Qin et al. (2017) proved that native dialect effects exist in the use of acoustic cues in English lexical stress by Standard Mandarin, Taiwanese Mandarin, and English speakers. Their findings showed that Standard Mandarin-speaking learners explored more duration than Taiwanese Mandarin-speaking learners to perceive English non-words. The reason is that standard Mandarin has neutral tone, but the Taiwanese Mandarin does not. Native dialect, it is suggested, is crucial for determining whether L2 learners can utilize some prosodic cues in English stress perception.

The literature review reported conflicting results about non-native learners who used the acoustic parameters in producing English stress. Cross-dialect studies of English lexical stress production remain scarce, and few research has compared the acoustic characteristics of F0, duration, and intensity simultaneously across different dialects.

### The Present Study

#### Research Questions

1. To what extent do different Chinese dialect (Beijing, Changsha, and Guangzhou) speakers produce F0, duration, and intensity patterns which deviate from American English speakers?
2. Whether, and if so, how, does Chinese dialects affect L2 speaker production of English lexical stress?

#### Hypotheses

1. Previous studies of L2 production have shown that language transfer effects from L1 to L2 is robust (Ploquin, 2013). As predicted by STM, Chinese dialect groups may show different patterns for the three acoustic correlates from the American English group.
2. As predicted by CAH, L2 dialect speakers are adept at using acoustic correlates in L2 if these correlates are actively used in L1 (i.e., F0 and intensity), whereas an inactive cue (i.e., duration) would cause difficulty for Guangzhou speakers, but not Beijing or Changsha speakers.

## Materials and Methods

### Participants

Eighty participants were recruited and constituted into four groups: American English, Beijing, Changsha, and Guangzhou groups (10 females and 10 males in each group). American English participants were US undergraduates. The Chinese speakers’ English proficiency was evaluated by *Language Experience and Proficiency Questionnaire* (Marian et al., 2007) and the *Lexical Test for Advanced Learners of English* (Lemhöfer and Broersma, 2012). Chinese participants were all roughly at the intermediate English proficiency levels. No participants reported being diagnosed with any cognitive or speech disorders. The self-evaluation of English proficiency of the participants was in Supplementary Table 1. Participants’ demographics were listed in Supplementary Table 2.

### Stimuli

Five minimal stress pairs were selected following the methodology of Fry (1955), Zhang et al. (2008), and Lai (2008). All stimuli were disyllabic English stress minimal pairs (Table 1). The stress patterns (trochee and iamb) of the stimuli were counterbalanced across participants. The target words were embedded in context sentences and carrier sentences. Target words were embedded in a carrier sentence using the pattern: “Please say ‘X’ **CLEARLY** but not **LOUDLY**” (Chan, 2007). This manipulation was designed to let prominence-lending pitch movements fall on the two words (“clearly” and “loudly”) and to reduce the chances that the target words “X” would receive the highest level of prominence in the sentence. Only the word in isolation was analyzed.

**Table 1:** Target words and context sentences.

Stimuli	FREQ^a^	Noun/Verb	Context sentence
**UP**set	17,592	Noun	I had a stomach **UP**set last night.
up**SET**		Verb	The rain has up**SET** our plans for a picnic.
**PRE**sent	75,339	Noun	We got a nice **PRE**sent from her.
pre**SENT**		Verb	She will pre**SENT** the awards to all.
**IM**pact	56,629	Noun	His father has a strong **IM**pact on him.
im**PACT**		Verb	The new policy will strongly im**PACT** the stock market.
**OB**ject	22,728	Noun	What is the **OB**ject on the table?
ob**JECT**		Verb	They will not ob**JECT** to your decision.
**SUB**ject	57,152	Noun	The latest **SUB**ject is education reform
sub**JECT**		Verb	We are sub**JECT** to many influences.

a *FREQ: Word frequency determined by the Corpus of Contemporary American English (COCA). Low-frequency words were not used*.

### Recording of Stimuli

All recordings were digitized using SENNHEISER PC 166 headset recorders with 44.1 kHz sampling rate and in a 16-bit rate mono format. The microphone was placed approximately 20 cm from the participant’s lips at an angel of 45° (horizontal). Recordings of stimulus were taken in a quiet room with the software Cool Edit Pro 2.0. The starting and ending points of each sound wave were selected at zero-crossing in order to prevent clipping artifacts. Each recording was then saved as an individual sound file.

### Procedure

The participants were invited to fill out a language background questionnaire and sign the written informed consent to participate in the study. A brief introduction of the experiment was presented to ensure that all the participants know how to participate in this experiment. Prior to the formal recording process, 5 minutes was given for participants to acquaint themselves with the experimental instructions, stimuli, and procedures. Then, they were asked to do a real word familiarity rating task. The familiarity scale options are divided into three levels: 1 = not at all familiar; 2 = A little familiar; and 3 = very familiar. These stimuli all obtained high familiarity score: 3. There was no difference in rating the word familiarity among the four groups. Stressed syllables were in capitalized and bolded to clue participants to produce the correct stress patterns. Participants practiced on five, non-test, and stress pairs.

During recording, participants would first read the context sentence, then the carrier sentence, and finally, the target stimulus in isolation three times at normal speech rates. The participants should be familiar with this rule of stress shift to distinguish noun from verb for some English stress pairs. These stimuli were presented on the computer screen with Microsoft PowerPoint. Participants sit in front of the computer and control the speed of slide switching by pressing the space bar. They were allowed to re-record when a mistake was made. First, the stimuli were displayed below with the corresponding context sentence and carrier sentence at the top. Second, the target stimuli and corresponding context sentences were displayed. Third, only the stimuli were shown. The average running time for the whole experiment was about 30 min for each participant.

### Acoustic Measurements

Similar to the method used by Nguyen and Ingram (2005) and Lai (2008), the acoustic parameters were measured of vowels by using Praat (version 5.3.76; Boersma and Weenink, 2014) scripts: average F0 (Hz); duration (ms); and intensity (dB). As recommended in the Praat manual, F0 range was set depending on the gender of the speaker (male: 75–300 Hz; female: 100–500 Hz).

The acoustic data were auto-segmented by Prosogram (Mertens, 2004) and then manually segmented using waveform and spectrogram information based on the criteria described by Peterson and Lehiste (1960), Stevens (2002), and Zhang et al. (2008). For each token, a text grid was generated and four boundaries were determined, for example, onsets and offsets of the stressed and unstressed vowels. During the manual segmentation process, the first zero-crossing point of the first glottal pulse of the vowel (can be clearly shown in the enlarged waveform) was marked as the start point of the vowel and the last zero-crossing point of the last glottal pulse of the nasal murmur as the end point of the nasal murmur. Two examples of the segmentation criteria for noun and verb were in the Supplementary Figures 1, 2.

F0 contours were obtained by taking 11 points (in Hz) in the rhyme part of each vowel by using ProsodyPro (Xu, 2013), http://www.homepages.ucl.ac.uk/~uclyyix/ProsodyPro/. Note that during each sonogram, the visible pitch contour (the short-dotted line) does not run through exactly from the beginning to the end of the interval. This is because pitch at some section cannot be defined by the Praat, but the missing pitch section is still considered as part of the sonorant. The undefined pitch will be ignored by pitch-based measurement, so it does not affect the final results.

The elicitation procedure yielded 2,400 tokens (5 words × 2 stress pattern × 3 repetitions × 80 participants = 2,400). Using Zhang et al. (2008)’s method, an acoustic analysis was made on the token that “each production is assumed to represent the speaker’s best attempt to produce stress on the appropriate syllable.” Participant best attempts to produce stress on the appropriate syllable were analyzed. The 800 tokens produced in isolation were used for analysis. The two vowels of each token were extracted for F0, duration, and intensity. Thus, the total trial data amount should be 1,600 (five words × 2 stress pattern × 2 vowel × 80 participants = 1,600). This study used 1,510 data for the subsequent analysis. Ninety trial data were excluded from further acoustic analysis. The reasons for exclusion were as: (1) the recording quality of three American participants did not meet acoustic extraction requirements. Their data were excluded (3 speakers * 2 stress * 5words * 2 vowels = 60). (2) Another 30 data were excluded due to incorrect stress patterns or unclear recordings. There were 1,510 duration data and 1,510 intensity data. There were only 1,474 F0 data. There were 36 missing F0 values in the process of extracting acoustic parameters using Praat. This absence may be caused by phonation-caused F0 curve fracture. All F0 contours were plotted based on 10 (words) × 80 (speaker) samples. Each curve is an average of 5 (words) × 20 (speakers). F0 contours were measured at every 10% of the contours at normalized time points and plotted by R Core Team (2013).

### Reliability

The intra- and inter-judge reliability assessment were examined for the acoustic measurement of F0, duration, and intensity by calculating extracted 10% random samples. The selected samples were measured by the same phonetician a second time for intra-judge reliability measure. Then, the speech samples were measured by another trained phonetician again. The mean absolute errors for F0, duration, and intensity for intra-judge measurement were 3.85 Hz, 10.21 ms, and 0.45 dB, respectively. For F0, duration, and intensity, the *Pearson*’s *linear correlation coefficients* between the first and second measurements were *r* = 0.94, *r* = 0.84, and *r* = 0.97, respectively. For F0, duration, and intensity, the mean absolute errors for inter-judge measurement were 4.1 Hz, 12.01 ms, and 1.81 dB, respectively. Inter-judge reliability for F0 was: *r* = 0.90, for duration: *r* = 0.89, and for intensity: *r* = 0.94.

### Statistical Methods

The statistical analysis method follows the previous studies (Zhang et al., 2008; Saha and Mandal, 2015). Four (group) * 2 (stress) mixed factorial ANOVAs were conducted on originally measured values (F0, duration, and intensity), with language group (American English, Beijing, Changsha, and Guangzhou) as between-subject variables, stress pattern (stressed or unstressed) as the within-subjects factor. *Post-hoc* (Tukey HSD) tests analyzed the parametric differences between the groups. All *post-hoc* tests were conducted with *p* = 0.05. One-way ANOVAs compared group S/U differences. Statistical analyses were conducted with R Core Team (2013), with *p* = 0.05 as a standard level of significance.

### Acceptability Ratings

The participants’ ratings of acceptability or accent are usually applied in determining their participant’s foreign accent (Flege, 1988; Piske, 2012). The common strategies to measure the level of foreign accent are used by inviting Native American English listeners to assign a numeric value to the utterances of the speakers based on its perceived quality. In the present study, to evaluate the acceptability of each stimulus, a listening evaluation task was conducted. Three linguistically trained phoneticians served as the consultants. They have to judge the foreign accent of the recordings of Chinese dialect participants (see Supplementary Table 3). They were told to focus on stress characteristics in rating process rather than other segmental.

The recordings were played randomly but blocked by gender. The raters first heard the recording of the target words and were asked to determine which word they heard. The two possible choices for each token (e.g., **SUB**ject or sub**JECT**) were shown on the screen until they made a choice. The raters were invited to rate the tokens produced by the four language groups on a five-point scale (1 = poor and 5 = excellent). The tokens and recordings were presented by using E-prime version 2.0 (Schneider et al., 2002).

## Results

**Figure 2:**
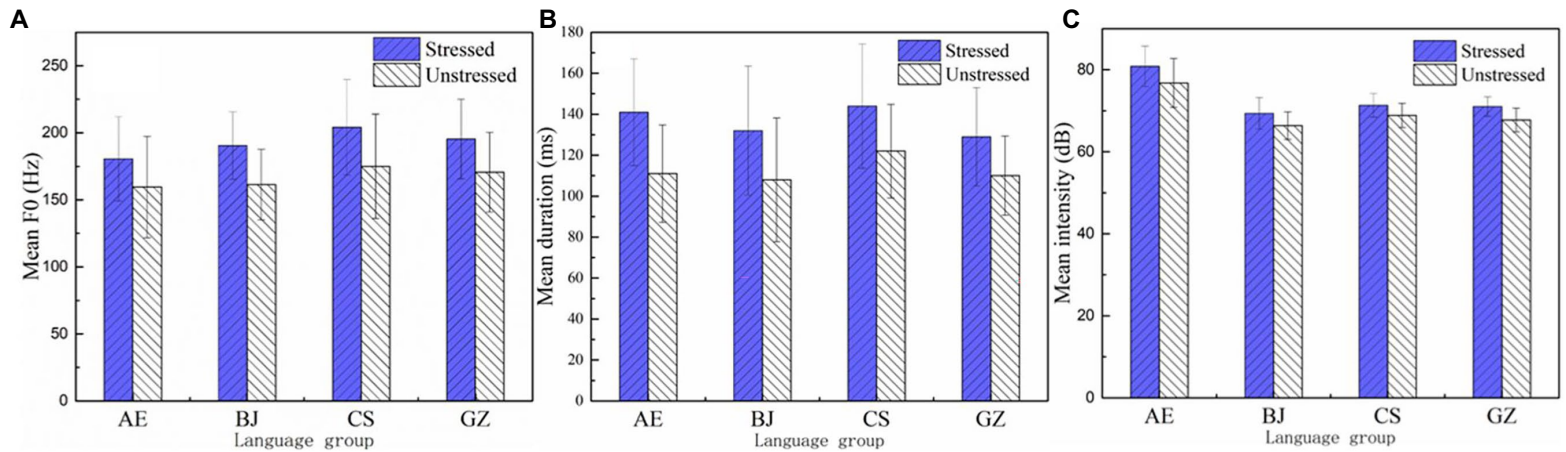
Mean values of F0 **(A)**, duration **(B)**, and intensity **(C)**.

**Figure 3:**
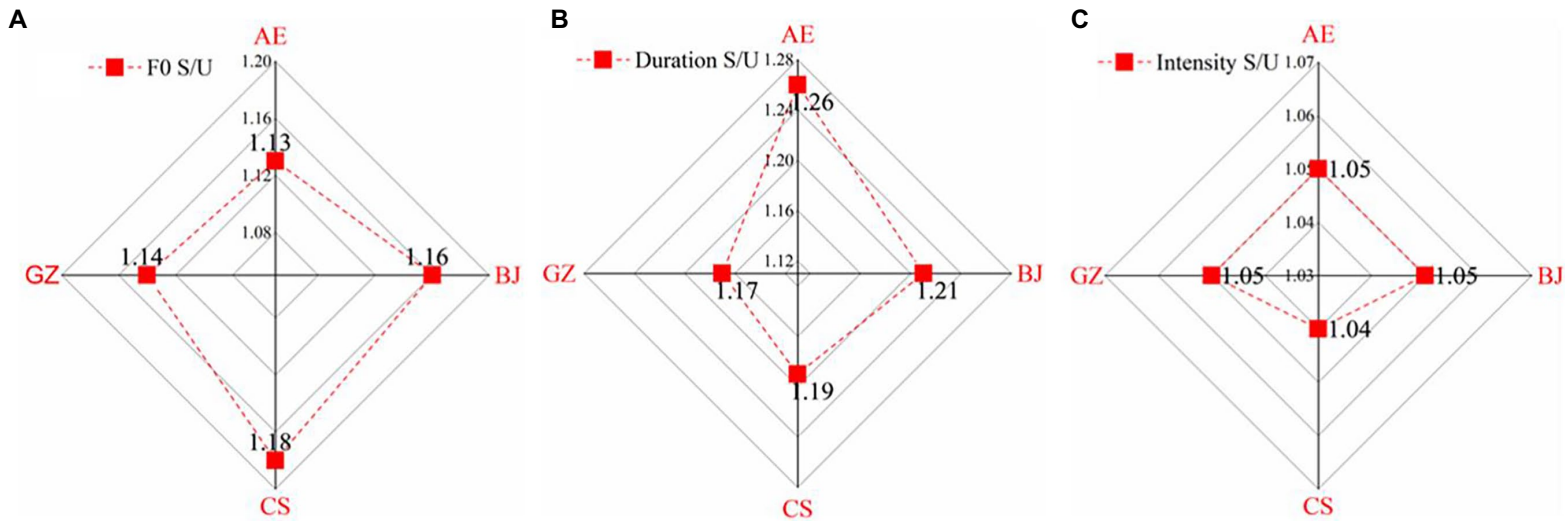
Average S/U ratios of F0 **(A)**, duration **(B)**, and intensity **(C)**.

Using absolute values (Figure 2) and stressed-to-unstressed vowel (S/U) ratios (Figure 3) of F0, duration, and intensity as indices, this study explores how American speakers and Chinese dialect speakers marked prosodic targets. ANOVA results of the mean values (Table 2) and S/U ratios (Table 3) were summarized.

**Table 2:** ANOVA summary of mean F0, duration, and intensity.

Acoustic cues	Source	*SS*	*df*	*MS*	*F*	*p*	$\eta_{p}^{2}$
F0	Group	136,377	3	45,459	12.185	<0.001^***^	0.024
	Stress	92,481	1	92,481	24.789	<0.001^***^	0.017
	Group × Stress	2,156	3	719	0.193	0.901	0.000
	Error	5,469,210	1,466				
Duration	Group	44,907	3	14,969	11.573	<0.001^***^	0.023
	Stress	50,725	1	50,726	39.218	<0.001^***^	0.025
	Group × Stress	14,737	3	4,912	3.798	0.010^**^	0.008
	Error	1,942,748	1,502	1,293			
Intensity	Group	7,755	3	2,585	107.595	<0.001^***^	0.177
	Stress	2,845	1	2,845	118.407	<0.001^***^	0.073
	Group × Stress	62	3	20.607	0.858	0.462	0.002
	Error	36,084	1,502	24.024			

*** *p* ≦ 0.001;

** *p* ≦ 0.01;

*p* > 1.

**Table 3:** ANOVA summary of S/U ratios of F0, duration, and intensity.

Acoustic correlate	*df*	*F*	*p*	$\eta_{p}^{2}$
F0	3	4.283	0.005^**^	0.018
Duration	3	5.487	0.001^***^	0.021
Intensity	3	0.702	0.551	0.168

*** *p* ≦ 0.001;

** *p* ≦ 0.01;

*p* > 1.

### F0

Mean F0 results appear in Figure 2A. Factorial ANOVA revealed significant main effects of group [*F* (3, 1,466) = 12.185, *p* < 0.001, $\eta_{p}^{2}$ = 0.024] and stress [*F* (1, 1,466) = 24.789, *p <* 0.001, $\eta_{p}^{2}$ = 0.017], but not group × stress interaction [*F* (1, 1,466) = 0.193, *p* = 0.901, $\eta_{p}^{2}$ = 0.000]. *Post-hoc* tests showed American English speakers produced a significantly different mean F0 from Changsha (*p* < 0.001) and Guangzhou (*p* = 0.005), but not from Beijing speakers (*p* = 0.110). Significant mean F0 differences were found in Beijing vs. Changsha speakers (*p* = 0.036) and Changsha speakers vs. Guangzhou speakers (*p* = 0.001), but not in Beijing vs. Guangzhou speakers (*p* = 0.762).

The three dialect groups produced greater S/U ratios of F0 than American English group (Figure 3A). Ranking order was as: Changsha (1.18) > Beijing (1.16) > Guangzhou (1.14) > American speakers (1.13). One-way ANOVA showed significant main effects for group [*F* (1, 716) = 4.283, *p* = 0.005, $\eta_{p}^{2}$ = 0.018]. *Post-hoc* tests showed the American group differed significantly from the Changsha group (*p* = 0.002), but not from Beijing or Guangzhou (*p >* 0.05). No significant differences were found among the dialect groups (*p* > 0.05).

### F0 Contour

**Figure 4:**
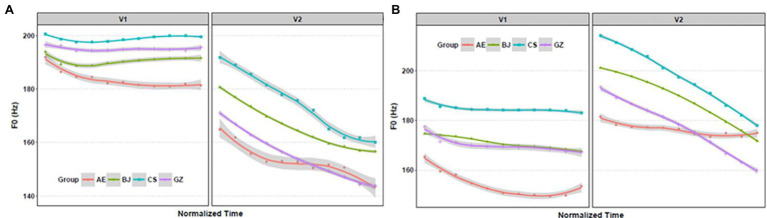
F0 contours in trochaic **(A)** and iambic **(B)** stress patterns for the four groups.

Figure 4 shows F0 contours in trochaic (a) and iambic (b) stress patterns.

Figure 4 reveals that contours for all groups differed in overall shape and height. Generally, F0 contours in the stressed vowels were higher than unstressed vowels. In the trochaic pattern, all dialect groups used high level F0 contours to signal-stressed vowels (V_1_). The American English group showed a gradual falling contour. All contours displayed declination for unstressed vowels (V_2_). F0 contours of the three dialect groups for stressed vowels (V_2_) in the iambic pattern displayed a high-falling tonal contour. The American English group contour was much less steep. All three dialect groups showed relatively more level contour patterns for unstressed vowel (V_1_) than the American English group.

Chinese dialect speakers are less flexible in realization of F0 contours. Moreover, they appeared to apply more fixed F0 contours in stressed syllables. When producing the stressed syllables in iambic, Chinese dialect speakers showed high-falling tonal contours with great F0 variation between the onset and offset. Moreover, the contours generated by the Chinese dialect speakers showed a rise/plateau at the onset point, which resembles the profile of the Chinese high-falling tone. In contrast, American speakers produced a relatively level F0 contour. Both in the trochaic and iambic stress pattern, the Chinese dialect groups showed a greater F0 drop between the onset and offset; whereas the American group produced more flattened F0 contours.

When producing the unstressed syllables, F0 contours all displayed a gradual falling trend. The F0 patterns are relatively consistent across the four groups. The F0 contours resembled the high-falling tone 4 in Mandarin. In contrast, the F0 contours of the American group showed relatively flat with a gradual falling trend. Generally, the F0 contours of the stressed syllables in trochaic stress pattern produced by the Chinese dialect speakers carry a high-level contour, while the stressed syllables in iambic stress pattern show a high-falling tonal contour.

F0 contours of each participant were shown in Supplementary Figure 3. To better understand the varying nature of the F0 contour over the time course of the stressed and unstressed syllable, the repeated measures General Linear Model ANOVAs were conducted. All statistical analyses were conducted by R Core Team (2013). Results of repeated measures ANOVA showed main effects for language group (*p* < 0.05). *Post-hoc* tests showed that in trochaic pattern (V_1_), there was a significant difference between the Beijing and Changsha groups (*p* < 0.05), Beijing and Guangzhou groups (*p* < 0.05); in trochaic pattern (V_2_), there was a significant difference between Beijing and Changsha groups (*p* < 0.05); significant differences were found between the American and Changsha groups from point 1–6 (*p* < 0.05), but not point 7–11 (*p* > 0.05); in iambic pattern (V_1_), there were significant differences between the Beijing and Changsha groups (*p* < 0.001); and the American and Changsha groups from point 3–11 (*p* < 0.05), but not point 1–2 (*p* > 0.05). In iambic pattern (V_2_), a significant difference was found between the American and Changsha groups (1–7 points; *p* < 0.05), but not 8–11 point (*p* > 0.05). No significant differences were found among the other groups in the four conditions.

### Duration

Mean duration results appear in Figure 2B. A factorial ANOVA revealed significant main effects of group [*F* (3, 1,502) = 11.573, *p* < 0.001, $\eta_{p}^{2}$ = 0.023], stress [*F* (1, 1,502) = 39.218, *p* < 0.001, $\eta_{p}^{2}$ = 0.025], and group × stress interaction [*F* (1, 1,502) = 3.798, *p* = 0.01, $\eta_{p}^{2}$ = 0.008]. *Post-hoc* tests showed the American English speakers produced significantly different duration values from Changsha (*p* = 0.042) and Guangzhou speakers (*p* = 0.014), but not Beijing speakers (*p* = 1.000). Significant duration differences were found in Beijing vs. Changsha (*p* = 0.028); Beijing vs. Guangzhou (*p* = 0.009); and Changsha vs. Guangzhou groups (*p <* 0.001).

The three dialect groups produced smaller S/U duration ratios for the American English group (Figure 3B): American English (1.26) > Beijing (1.21) > Changsha (1.19) > Guangzhou (1.17). One-way ANOVA showed significant main group effects [*F* (1, 753) = 5.487, *p* = 0.001, $\eta_{p}^{2}$ = 0.021]. *Post-hoc* tests showed the American English speakers differed significantly from Guangzhou speakers (*p* = 0.022), but not from Beijing or Changsha speakers (*p* > 0.05). Significant differences were found in Beijing vs. Changsha (*p* = 0.003); Beijing vs. Guangzhou (*p* = 0.001); and Changsha vs. Guangzhou groups (*p* = 0.044).

### Intensity

Mean intensity results appear in Figure 2C. Factorial ANOVA revealed significant main effects of group [*F* (3, 1,502) = 107.595, *p* < 0.001, $\eta_{p}^{2}$ = 0.177] and stress [*F* (1, 1,502) = 118.407, *p* < 0.001, $\eta_{p}^{2}$ = 0.073], but not group × stress interaction [*F* (1, 1,502) = 0.858, *p* = 0.462, $\eta_{p}^{2}$ = 0.002]. *Post-hoc* analysis showed American speakers produced significantly larger mean intensity values than either Beijing, Changsha, or Guangzhou speakers (*p* < 0.001). Significant mean intensity differences were found in Beijing vs. Guangzhou (*p* = 0.001), Changsha vs. Guangzhou (*p* = 0.004), but not in Beijing vs. Changsha speakers (*p* = 0.989).

Figure 3C reveals that dialect groups produced S/U intensity ratios similar to the American group. One-way ANOVA showed no significant main effects of group [*F* (1, 752) = 0.702, *p* = 0.551 $\eta_{p}^{2}$ = 0.168]. *Post-hoc* tests showed that American speakers showed no significant difference with Beijing speakers (*p* = 0.706), Changsha speakers (*p* = 0.914), and Guangzhou speakers (*p* = 0.518). No significant difference was found among Chinese dialect groups: Beijing vs. Changsha (*p* = 0.972); Beijing vs. Guangzhou (*p* = 0.989); and Changsha vs. Guangzhou (*p* = 0.875).

## Discussion

### L1 Prosodic Effects on F0

F0 results showed the Beijing and Guangzhou speakers F0 ratios did not differ significantly from American English speakers. This suggests that an active F0 role as L1 dialect tonal cues facilitated production of F0 contrasts. These results are consistent with Choi et al. (2016). They found L1 lexical tone facilitated L2 English lexical stress sensitivity due to a direct prosodic transfer. Chinese learners interpret English stressed or unstressed differences as tone differences (Cheng, 1968).

Changsha speakers’ F0 S/U ratios were significantly different from the American English speakers. Juffs (1990) found the Changsha dialect speakers usually realize stress by “extending the length of the syllable to an inordinate degree,” but “with no pitch movement,” and “the syllable was in fact tonic.” Changsha speakers usually failed to assign the lexical stress correctly *via* simple increases in F0 height without movement (Juffs, 1990). Some Changsha speakers might have applied F0 movement indiscriminately to English stress.

The Guangzhou speakers could differentiate the F0 contrasts. Chen and Au (2004) reported Cantonese speakers habitually assign high-level tones (/55/) to the primary stress in English, while use tone regularity (/22/ and /11/) to assign the less prominent syllable. For example, they use Cantonese Tone /22–55/ to assign English word /rɪ’zʌlt/ and /55–11/ to assign /'peɪpə/. Chinese dialect speakers may unconsciously use L1 tonal systems to assign English lexical stress.

### L1 Prosodic Effects on F0 Contour

American group F0 contours were more diverse than the three Chinese dialect groups. F0 contour results indicate that the Chinese dialect speakers employed higher F0 to signal-stressed syllables in a way similar to Chinese tonic pitch contours. F0 contours for stressed vowels (in trochee) for all Chinese dialect speakers resemble Mandarin’s high-level tone 1 (T1). Stressed vowel contours, in iamb, resemble the high-falling tone 4 (T4) in Mandarin, consistent with Lai and Sereno (2008). Level tone is rare in English (Roach, 1983), but common in Chinese languages. Juffs (1990) observed that Chinese students use T1 with a higher pitch for English lexical stress due to an over-generalization from L1.

F0 contours of the American speakers were more diverse than Chinese speakers. Results suggest that Chinese dialect speakers tend to use more fixed contours in stressed syllables; whereas American English speakers F0 contours are more flexible than Chinese dialect speakers. This divergence may result from the fact that the Chinese dialect speakers’ inflexible usage of F0 cues in L1 dialects, since the tone patterns are fixed in Chinese (Lai, 2008). This fixedness may result in dialect speakers using F0 contour inflexibly. Besides, the F0 phonemic status triggers a more consistent use of a cue due to the F0 phonemic feature in Chinese. Zhang et al. (2008) found that some Chinese speakers produced non-English-like F0 patterns, with a non-standardly F0 values in stressed syllables. Chinese learners usually use the common strategy—tone assignment in L2 stress production (Chen and Au, 2004).

Interestingly, the three Chinese dialect speaker groups exhibited obvious differences. Changsha dialect speakers showed higher pitch contour for stressed and unstressed syllables. F0 of the neutral tone in Changsha dialect is level (i.e., mid-level tone; Zhang, 2005). Prior studies showed that “high tones predominate in most dialects,” and “the falling tone occurs more frequently than any other tonal contour” (Cheng, 1973). Different Chinese dialects have different tonal inventories resulting in various F0 contour patterns. For example, F0 contours of the Changsha speakers are similar to the high-level tone in Changsha dialect, suggesting that Changsha speakers may adopt either the High tone (High level 1, 45) or the Falling tone (41). Beijing speakers may apply either the High tone (High level 1, 55) or the Falling tone (51). GZ speakers may apply either the High-level tone (High level 1, 55) or the Falling tone (21). Chinese learners use high tones (high level or falling) to signal-stressed syllables in English; whereas native English speakers do not. Statistical methods for analyzing F0 contours, such as orthogonal polynomials, growth curve analysis, and functional data analysis, for example, those recently used by Chen et al. (2017), merit further study.

### L1 Prosodic Effects on Duration

Duration differences between American and Chinese dialect speakers may occur for any one, or a combination of, the following reasons. English syllables are stress-timed while Chinese syllables tend to be syllable-timed. Stress-timed languages tend to vary in duration more than syllable-timed languages (Mok, 2009). Chinese is monosyllabic and its longer words are composed of independent monosyllables. Each Chinese syllable carries a tone and “each tone is more or less of equal length” (Chen, 2015). This may result in Chinese dialect groups’ smaller S/U ratios of duration than American speakers, but the statistical results showed that only American speakers differed significantly from Guangzhou speakers.

American English speakers produced significantly different S/U duration ratios from Guangzhou speakers, but not Beijing or Changsha speakers. The reasons may be due to that the unstressed syllables (neutral tone) occur more frequently in Beijing dialect and Changsha dialect, but not in the Guangzhou dialect. Unstressed syllable duration was dramatically shortened (Brainerd and Chao, 1968). The Guangzhou dialect has no neutral tone and maybe not duration sensitive. Beijing and Changsha speakers perform better when using duration than Guangzhou speakers. Different duration proportions between Guangzhou speakers and American English speakers suggest negative L1 transfer effects. Guangzhou speakers might have difficulties producing the requisite vowel duration contrasts for English lexical stress.

### L1 Prosodic Effects on Intensity

S/U intensity ratios for each group approximated 1.0 indicating minimal intensity differences for the stressed and unstressed vowels. The ratios of the three dialect groups were similar to that of the American group. This suggests that the three Chinese dialect groups produced intensity contrasts natively. Zhang et al. (2008) proved that Mandarin speakers can successfully use intensity to produce English stress in a native-like fashion. Whalen and Xu (1992) concluded that intensity was the extra-essential cue for Mandarin word stress.

In sum, the results are compatible with the predictions of this study. When L2 dialect speakers did not express these acoustic cues in the same way as those from the American group, the performance pattern was consistent with the characteristic transfer of the dialect tonal system. Results generally confirmed the two hypotheses. The findings tended to prove that the prosodic structure and rhythmic typology are inherent to a given language or dialect, and they are deeply embedded in the learners’ language-specific competence, which makes it difficult for L2 learners to get rid of, results in a foreign accent. This is evidence of language transfer.

## Conclusion

Results suggest that speakers of these three Chinese dialects produced less native-like stress patterns, although they used the three cues to distinguish stress. Chinese dialect speakers showed some divergence from American speakers in a manner consistent with the transfer of characteristics of L1 dialects. Beijing speakers differentiated English stress contrasts in terms of F0, duration, and intensity, suggesting active F0 and intensity roles, as tonal cues in Beijing dialect might facilitate producing F0 and intensity contrasts. Beijing and Changsha dialect speakers were adept in using duration as it was actively used in signal neutral tones. Changsha speakers produced native-like duration and intensity contrasts but showed significant F0 differences. Guangzhou speakers produced native-like F0 and intensity contrasts but failed to produce duration contrasts due to a negative transfer effect. Duration does not function as an active cue in Guangzhou tonal distinctions. As predicted by STM, and CAH, the results suggest that L1 dialect would transfer to the production of L2 lexical stress. Future research is needed to provide additional insights into how the prosodic features of other native dialects are transferred into L2 prosody production.

This study would contribute to general second language acquisition studies both in theory and in practice. The study will provide evidence to show how non-native English speakers acquire the stress. It is hoped that the findings of the present study can be implemented in language teaching classes, language recognition, or other language mediums, such as speech clinics.

Some limitations need to be acknowledged. First, a linear mixed-effects model analysis with item-level information would be more informative than ANOVA analysis. Second, in addition to F0, duration, and intensity, vowel reduction is also an important acoustic cue for English lexical stress. The use of vowel reduction by learners with dialect background in English stress is worth further exploration. Third, three repetitions of the tokens produced in the manuscript should be analyzed instead of choosing to the production of the best attempt. Fourth, this study only involves three dialects. In the future, it is necessary to study the English stress production by the speakers from other dialect areas. Fifth, the acoustic analysis of this study only analyzes isolated disyllabic words. It is interesting to explore whether L1 dialects show transfer effect in L2 spontaneous discourse.

## Data Availability Statement

The raw data supporting the conclusions of this article will be made available by the authors, without undue reservation.

## Ethics Statement

Ethical review and approval was not required for the study on human participants in accordance with the local legislation and institutional requirements. The participants provided their written informed consent to participate in this study.

## Author Contributions

XG proposed the research questions, designed the experiment, collected data, conducted statistical analysis, wrote the draft of the manuscript, and revised it. The author approved the submitted version.

## Funding

This research was funded by the Shanghai Philosophy and Social Science Planning Education Youth Project, 2019, grant number B19005.

## Conflict of Interest

The author declares that the research was conducted in the absence of any commercial or financial relationships that could be construed as a potential conflict of interest.

## Publisher’s Note

All claims expressed in this article are solely those of the authors and do not necessarily represent those of their affiliated organizations, or those of the publisher, the editors and the reviewers. Any product that may be evaluated in this article, or claim that may be made by its manufacturer, is not guaranteed or endorsed by the publisher.
