# Supplementary material for: Acoustic Correlates of English Lexical Stress Produced by Chinese Dialect Speakers Compared to Native English Speakers
Source: Front Psychol. 2022 Mar 8;13:796252. doi: 10.3389/fpsyg.2022.796252 (PMC8958030; doi:10.3389/fpsyg.2022.796252)
Supplement: Supplementary file 1 [file Table_1.docx]

Supplementary Material

## Supplementary Tables

## Table 1 Self-evaluation of English proficiency of three Chinese dialect groups (Guo and Chen, 2022)

|  | **Degree of practice with English** | | | **Mean** |
| --- | --- | --- | --- | --- |
|  | **BJ** | **CS** | **GZ** |  |
| Pronunciation (1: Extremely poor- 10: Perfect) | | | | |
| Mean | 6.35 | 6.17 | 6.03 | 6.18 |
| SE | 0.42 | 0.44 | 0.56 | 0.47 |
| Vocabulary (1: Extremely poor- 10: Perfect) | | | | |
| Mean | 6.89 | 6.43 | 6.98 | 6.77 |
| SE | 0.37 | 0.54 | 0.46 | 0.46 |
| Grammar (1: Extremely poor- 10: Perfect) | | | | |
| Mean | 6.87 | 6.41 | 6.73 | 6.67 |
| SE | 0.44 | 0.34 | 0.54 | 0.44 |

## Table 2. Participants’ Demographics (Guo and Chen, 2022)

| **Group** | **Gender (F/M)** | **Age^a^**  **(s.d.)** | **AOA^b^**  **(s.d.)** | **DEU^c^**  **(s.d.)** | **DMU^d^**  **(s.d.)** | **DDU^e^**  **(s.d.)** | **Proficiency^f^**  **(s.d.)** | **LexTALE^g^**  **(s.d.)** |
| --- | --- | --- | --- | --- | --- | --- | --- | --- |
| **BJ** | 10/10 | 20.2  (2.2) | 13.1 (2.5) | 14.1 (3.0) | 38.5  (0.8) | 47.4  (0.3) | 6.7  (1.2) | 66.4  (3.4) |
| **CS** | 10/10 | 17.2  (2.0) | 10.4  (2.2) | 11.3 (7.5) | 32.5  (2.1) | 56.2  (0.5) | 6.1  (2.3) | 60.7  (2.5) |
| **GZ** | 10/10 | 18.8  (1.9) | 10.2 (2.3) | 9.3 (4.3) | 31.7  (0.7) | 59.0  (0.2) | 5.8  (1.6) | 57.9  (2.3) |
| **AE** | 10/10 | 22.7  (2.9) | -- | -- | -- | -- | -- | -- |

s.d.: standard deviation.

^a^Age: mean age when testing (in years).

^b^AOA: mean age of English acquisition (in years).

^c^DEU: daily English usage (in percentage).

^d^DMU: daily Mandarin usage (in percentage).

^e^DDU: daily dialect usage (in percentage.

^f^Proficiency: the proficiency evaluated by themselves (10-point Likert scale, 0 = “none” ; 10 = “perfect”).

^g^ LexTALE score was converted to a percentage.

## Table 3 Results of the perceptual evaluation of real words

| **Stimuli** | **AE** | | **BJ** | | **CS** | | **GZ** | |
| --- | --- | --- | --- | --- | --- | --- | --- | --- |
|  | **Accuracy^a^** | **Avg^b^**  **(s.d.)^c^** | **Accuracy** | **Avg**  **(s.d.)** | **Accuracy** | **Avg**  **(s.d.)** | **Accuracy** | **Avg**  **(s.d.)** |
| UPsep | 1.00 | 4.99  (0.22) | 0. 97 | 4.78  (0.59) | 0.96 | 3.21  (0.43) | 0.93 | 3.24  (0.22) |
| upSEP | 1.00 | 4.89  (0.30) | 0.91 | 3.76  (0.47) | 0.94 | 2.76  (0.43) | 0.97 | 3.29  (0.30) |
| PREsent | 1.00 | 4.99  (1.00) | 0.96 | 3.40  (0.29) | 0.92 | 1.57  (0.43) | 0.93 | 2.81  (1.00) |
| preSENT | 1.00 | 4.89  (0.87) | 0.98 | 4.76  (0.53) | 0.90 | 2.22  (0.27) | 0.96 | 1.27  (0.87) |
| IMpact | 1.00 | 4.93  (0.76) | 1.00 | 4.09  (1.02) | 0.99 | 3.43  (0.13) | 0.97 | 3.39  (0.76) |
| imPACT | 1.00 | 4.78  (0.83) | 0.99 | 4.98  (0.73) | 0.90 | 3.17  (0.43) | 0.92 | 3.45  (0.83) |
| OBject | 1.00 | 4.92  (0.62) | 0.97 | 4.56  (0.66) | 0.92 | 1.89  (0.43) | 0.91 | 3.56  (0.62) |
| obJECT | 1.00 | 4.93  (0.56) | 1.00 | 4.53  (0.32) | 0.91 | 3.15  (0.43) | 0.93 | 3.35  (0.56) |
| SUBject | 1.00 | 4.99  (0.34) | 1.00 | 4.89  (0.21) | 1.00 | 4.35  (0.22) | 1.00 | 4.98  (0.34) |
| subJECT | 1.00 | 4.98  (0.40) | 1.00 | 4.33  (0.31) | 0.93 | 4.21  (0.17) | 0.96 | 4.75  (0.14) |

Note: Capital letters indicate stressed syllables, lower-case letters indicate unstressed syllables.

^a^Accuracy: proportion of correct identifications;

^b^Avg: mean ratings of acceptability;

^c^s.d.: standard deviation.

As shown in Table 3, the native American raters correctly identified the majority of the production by the four language groups. Mean identification accuracy score for American English, Beijing, Changsha, Guangzhou speakers was 1, 0.98, 0.94, and 0.95, respectively. Most American English speakers’ production scored higher than 4.00 while the Chinese speakers (generally less than 4.00) produced less than AE speakers. One-way ANOVA analysis showed that there was a significant rating difference among the four language groups (*p* < 0.05).

## Supplementary Figures


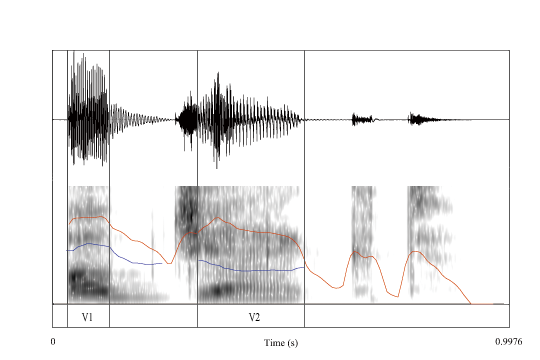


## Figure 1 An example of the acoustic measurements for the noun reading –OBject


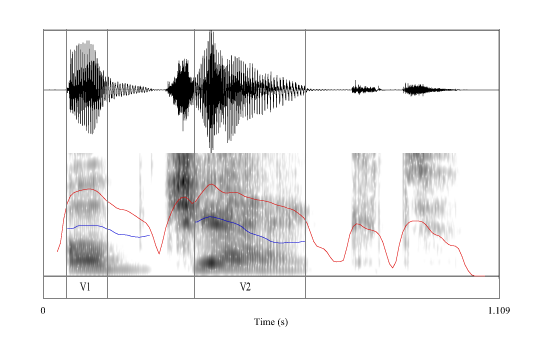


## Figure 2 An example of the acoustic measurements for the noun reading –obJECT

**F0 Contour**

The realization of F0 movements across the four groups was analyzed. In order to better evaluate the realization of F0 contours in each speakers’ production, Figure 3 indicates the F0 contour of each participant in the four language groups.


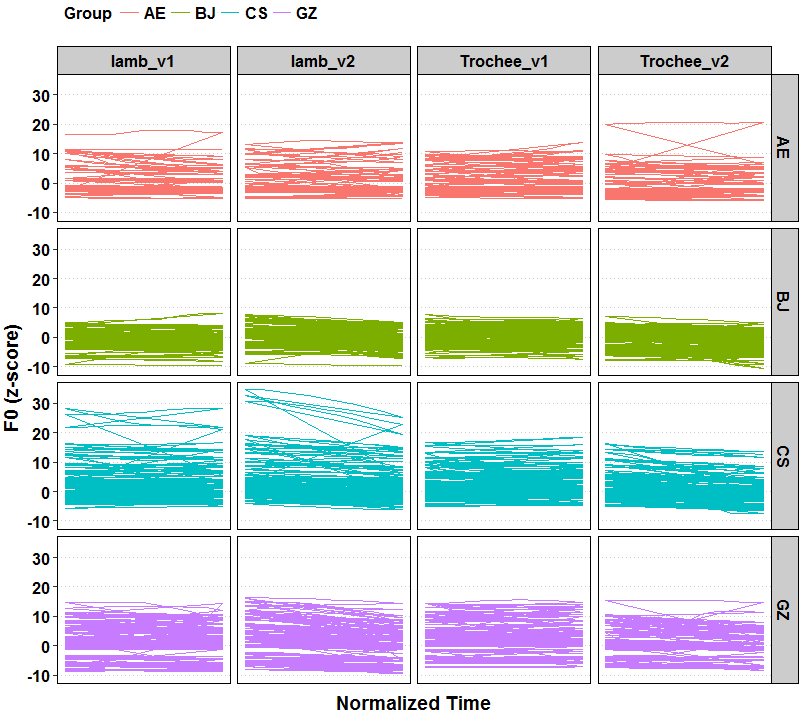


## Figure 3 F0 contours of each participant in four groups

As shown in Figure 3, speakers of the AE group and CS group show perturbation on F0 contours. But the BJ speakers showed the most consistent pattern in the four language groups.

## References:

Guo, X., and Chen, X. (2022). Perception of English Stress of Synthesized Words by Three Chinese Dialect Groups. *Front. Psychol.* 13:803008. Accepted. doi:10.3389/fpsyg.2022.803008
